# Secondary Myiasis in an Immunocompetent Patient With Severe Chromoblastomycosis

**DOI:** 10.7759/cureus.72733

**Published:** 2024-10-30

**Authors:** Adalina Torres, Christine E LeRoy, Bijal S Sheth, Sydney Weber, Kevin S Vasquez

**Affiliations:** 1 Family Medicine, St. Joseph's Regional Medical Center, Paterson, USA

**Keywords:** a case report, chromoblastomycosis, itraconazole, myiasis, treatment

## Abstract

This is the case of chronic chromoblastomycosis (CBM) in a 61-year-old male from the Dominican Republic (DR) with extensive cutaneous eruptions over multiple areas of the body including bilateral lower extremities and the flank extending to the back. A 61-year-old male with a history of morbid obesity, chronic kidney disease stage III, and well-controlled hypertension presented to the family medicine clinic for the evaluation of chronic painful skin lesions on his legs and torso. The lesions began 19 years prior, following a flood in the Dominican Republic (DR) where he was living at the time. He described the initial lesion as a dark flat spot that never healed, despite various home remedies. It began on his distal lower left extremity and slowly became more nodular and spread over his entire lower left extremity. Over the years, he continued to develop more nodular lesions on both legs and his left flank. Physical exam revealed multiple hyperkeratotic fungating nodules and papules on the bilateral lower extremities as well as the left flank. Physical exam of the left lower extremity showed multiple hyperkeratotic fungating nodular skin lesions. No biopsies were done at that visit. Three weeks after his initial presentation, the patient presented to the emergency department with complaints of bloody discharge and “maggots” in his leg wounds. On physical exam, there was evidence of two infected pedunculated lesions with surrounding erythema and maggots coming out of the wound on the left lower extremity. Using scissors, one of the lesions was excised, resulting in complete removal of the maggots. The tissue was sent to pathology for assessment and acid-fast bacilli (AFB) smear. Pathology report showed skin with marked papillary epidermal hyperplasia with prominent parakeratosis, pseudoepitheliomatous hyperplasia, granulation tissue, and extensive dermal acute inflammation with microabscesses. The larvae had a dense cuticle with viable internal organs and striated muscle. There was no evidence of malignancy, and AFB smear was negative. The patient was continued on Keflex and referred to infectious disease for further evaluation. Infectious disease started the patient on ivermectin 3mg four times a day due to the possibility of chronic larval migrans. A shave biopsy was performed by dermatology, and pathology showed marked papillary epidermal hyperplasia with parakeratosis and prominent pseudoepitheliomatous squamous hyperplasia. The dermis showed extensive chronic and focal acute inflammation with micro-abscesses and scattered multi-nucleated foreign body giant cells. Scattered within the pyogranulomatous inflammation in the dermis were singly and clusters of brown pigmented yeast forms, some engulfed by foreign body giant cells. Special stains (GMS and PAS-D) highlighted fungal organisms. These results in the context of extensive chronic lesions were compatible with chromoblastomycosis. Given this diagnosis, the decision was made to continue on itraconazole 200mg oral twice daily. Dermatology also began physical treatment with liquid nitrogen cryotherapy with topical Mupirocin ointment to be applied daily. This treatment is currently ongoing and shows significant decreases in severity of lesions as noted in decreased pain, significant flattening of the lesions. The patient is reassessed with repeat fungal cultures every six weeks.

## Introduction

Chromoblastomycosis is a subcutaneous fungal infection endemic to tropic and sub-tropic climates. It is primarily due to occupational exposure but can also occur after climate disasters in vulnerable populations [1]. This cutaneous fungus can be challenging to diagnose due to its chronic and indolent course, presenting in various forms including verrucous, nodular, tumoral, cicatricial, and plaque [2]. As demonstrated by this case, the diagnosis and treatment of chromoblastomycosis can be particularly challenging due to its variable presentation and slow progression. The disease is strongly associated with tropical climates and is more commonly found in individuals with exposure to organic matter and agriculture such as soil, plants, wood, and contaminated water [3].

Secondary myiasis is the infestation of necrotic or decaying tissue by fly larvae (maggots) in pre-existing wounds. Unlike primary myiasis, where larvae infest healthy tissue, secondary myiasis occurs in open, often infected wounds or sores that are already compromised. Secondary myiasis typically occurs in cases of poor hygiene or unsanitary conditions.

## Case presentation

**Figure 1:**
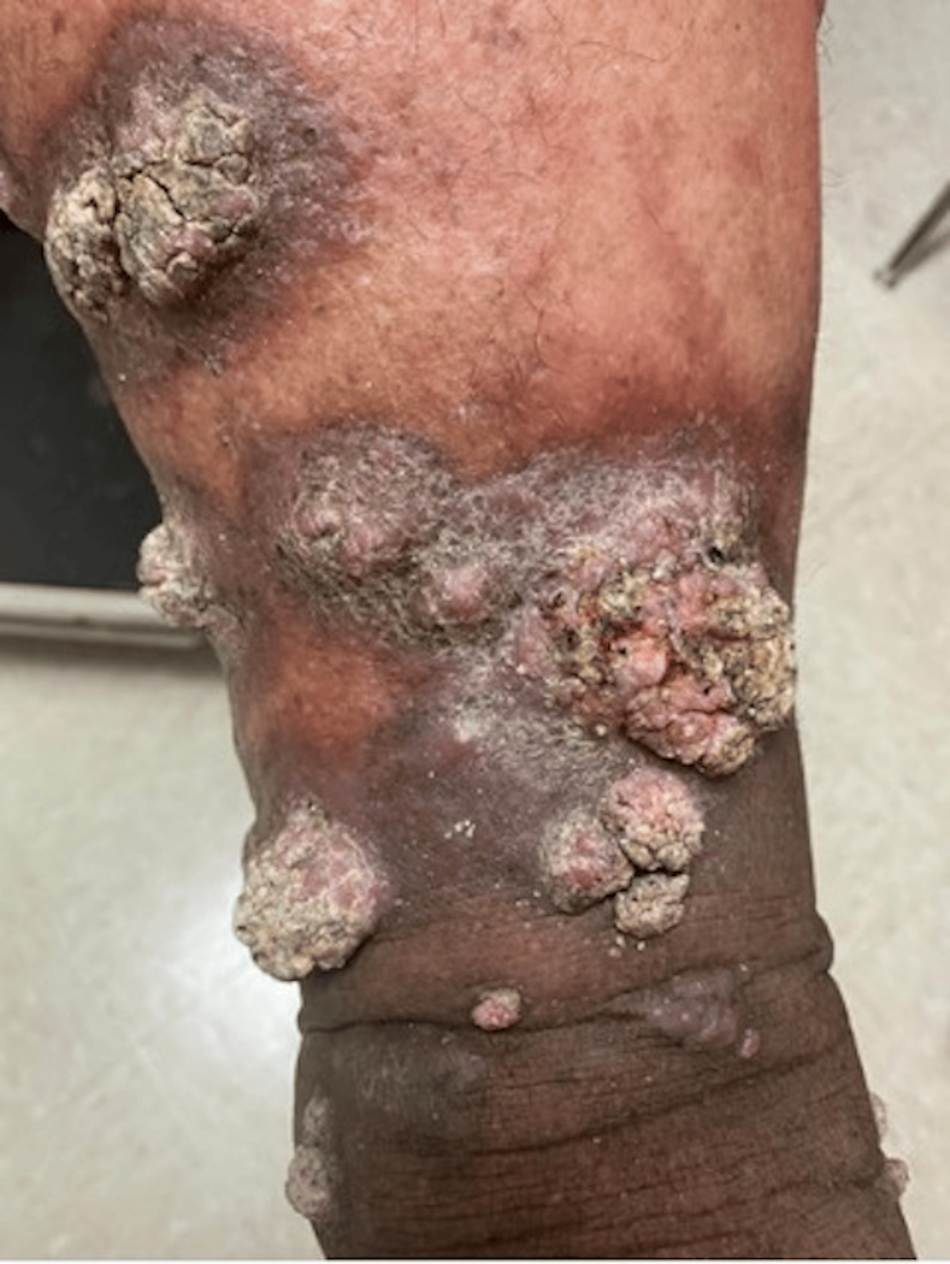
Multiple hyperkeratotic fungating nodules in upper extremities

**Figure 2:**
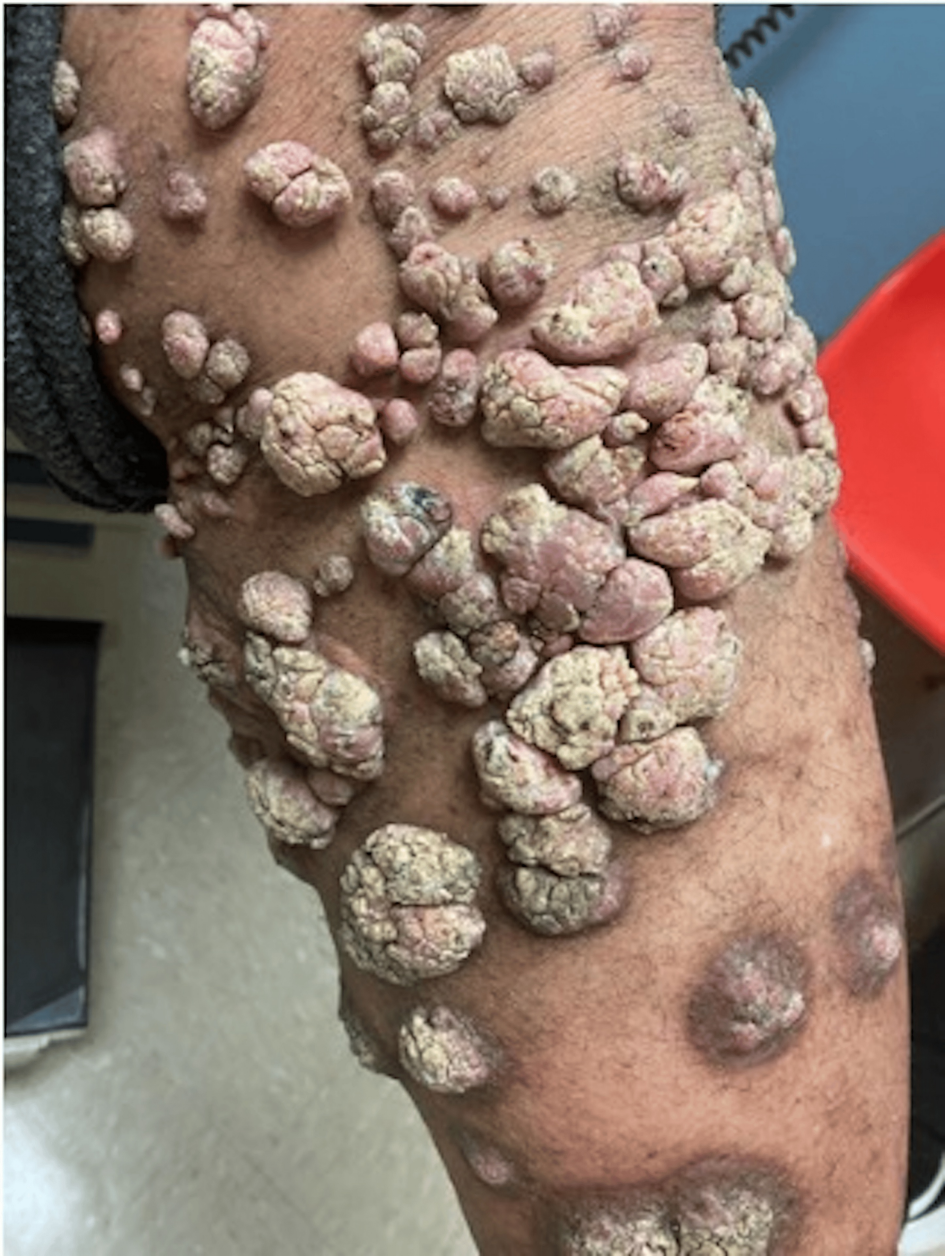
Multiple hyperkeratotic fungating nodules of lower extremities

A 61-year-old obese male with a history of chronic kidney disease and hypertension presented to the family medicine clinic with chronic painful skin lesions on his legs and torso. The lesions had originated 19 years ago following a flood in the Dominican Republic where he lived at the time. Starting as a non-healing dark spot on his left lower extremity, they gradually became nodular and spread to involve both legs and the left flank. Physical examination revealed hyperkeratotic fungating nodules and papules on his legs and left flank, as seen in Figures 1, 2.

Three weeks after his initial visit, the patient presented to the emergency department with complaints of bloody discharge and maggots emerging from his leg wounds. Physical examination confirmed infected pedunculated lesions with surrounding erythema. Surgical removal of maggots from one lesion was performed, and tissue sent for pathology showed marked inflammation and viable larvae. The patient was started on Cephalexin and subsequently treated with ivermectin for suspected chronic larval migrans.

A shave biopsy performed later confirmed chromoblastomycosis, revealing extensive chronic and acute inflammation with fungal organisms highlighted by special stains. Treatment was initiated with itraconazole and included physical therapies such as liquid nitrogen cryotherapy and topical Mupirocin ointment. This approach led to significant improvement in lesion severity, characterized by reduced pain and flattening of the lesions. The patient continues to be monitored with fungal cultures every six weeks to assess treatment response.

## Discussion

Chromoblastomycosis (CBM) poses diagnostic challenges, requiring a high index of suspicion among clinicians [4]. Diagnosis is often delayed, averaging 14 years, allowing lesions to progress and leading to complications such as secondary infections, lymphedema, myiasis, and occasionally malignant transformation, significantly affecting patients' quality of life [5-8]. When patients from tropical regions present with worsening nodular or verrucous skin lesions resistant to treatment, CBM should be considered early in the differential diagnosis.

Treatment typically involves physical interventions like cryotherapy or surgery, along with antifungal medications such as terbinafine or itraconazole, chosen based on patient tolerance and cost-effectiveness [6]. Long-term treatment spanning months to years may be necessary, especially for moderate to severe cases prone to recurrence and resistance [7,8]. CBM predominantly affects regions like Latin America, the Caribbean, Africa, and Asia, where delayed diagnosis can worsen outcomes, leading to treatment refractoriness and severe complications, including squamous cell carcinoma [8].

## Conclusions

This case of chronic chromoblastomycosis over the course of 19 years shows the clinical significance of early diagnosis and treatment on the patient's quality of life and prevention of complications. In the first two biopsies done for this patient, both were compromised by superinfection of myiasis. When patients report skin lesions and have a history of exposure to risk factors such as organic matter in areas endemic for CBM, there must be a thorough workup done, including histopathology with vigilance for the visualization of the characteristic pseudoepitheliomatous hyperplasia with muriform cells or sclerotic bodies.
